# Evaluation of bone health among epileptic patients using biochemical markers and DEXA scan: an Egyptian study

**DOI:** 10.1186/s41983-018-0014-2

**Published:** 2018-04-25

**Authors:** Ebtesam Mohamed Fahmy, Laila Ahmed Rashed, Rania Shehata Ismail, Hanan Helmy, Doaa Atef Mekkawy

**Affiliations:** Faculty of Medicine, Cairo University, Giza, Egypt

**Keywords:** Epilepsy, DEXA, AEDs, Vitamin D

## Abstract

**Background:**

An association between antiepileptic drugs, low bone mineral density (BMD), fractures, and abnormalities in bone metabolism has been suggested for a longer period, although conclusive evidence has not been reported.

**Methods:**

Thirty epileptic patients and 30 matched healthy subjects participated in the study. Measurements of serum levels of calcium, phosphorus, vitamin D, parathormone, and alkaline phosphatase were done for included subjects. Dual-energy X-ray absorptiometry (DEXA) scan was also performed.

**Results:**

Serum calcium, phosphorus, and vitamin D were significantly lower, whereas serum parathormone and alkaline phosphatase were significantly higher in epileptic patients compared to control subjects. Bone mineral density (BMD) abnormalities were detected in 22 patients (73.4%). A statistically significant difference in DEXA scan measurements at different regions was detected between epileptic patients and control subjects. Epileptic patients receiving enzyme inducer antiepileptic drugs (AEDs) had significantly lower serum (calcium, phosphorous, and vitamin D) and lower BMD values compared to those receiving enzyme inhibitors. Results of BMD were positively correlated with serum calcium, phosphorous, and vitamin D, while negatively correlated with serum alkaline phosphatase and duration of therapy.

**Conclusions:**

Abnormal bone health is common in epileptic patients. These abnormalities may be attributed to prolonged intake of AEDs especially enzyme inducers.

## Background

Epilepsy is a major public health problem affecting nearly 50 million people worldwide. Treatment with antiepileptic drugs (AEDs) is generally chronic and mostly long life (Valsamis et al. 2006). Some studies had showed that the fracture rate in epileptic patients is two to six times higher than the observed rate in the general population (Souverein et al. 2005). Previous studies found that fracture risk was higher for hepatic enzyme inducing AEDs such as phenytoin (DPH), carbamazepine (CBZ), and phenobarbital (PB) than non-inducing AEDs, while no significant effect was reported with the new AED and ethosuximide (Vestergaard et al. 2004). Moreover, the duration of AED treatment, polypharmacy, and gender difference have positive correlation with predicted bone loss (Oner et al. 2004).

A large number of biochemical abnormalities affecting bone metabolism had been reported in most of epileptic patients on AEDs. These abnormalities include hypocalcemia, hypophosphatemia, low biologically active vitamin D levels, and increase in parathormone (PTH) level (Hamed et al. 2014). These effects have been commonly seen with hepatic enzyme inducing AEDs which increase the catabolism of vitamin D resulting in secondary hyperparathyroidism. AEDs also inhibit the cellular response to PTH resulting in increasing bone remodeling (Pascussi et al. 2005).

Vitamin D supplementation has been recommended for all subjects using AEDs, particularly for those with low vitamin D levels and high risk of bone disease and/or with documented low BMD (Mikati et al. 2006; Holick et al. 2011).

This study was designed to evaluate bone health among epileptic patients and to identify possible risk factors for bone mineral density abnormality in such patients.

## Methods

### Participants

This case-control study was carried on 60 subjects (30 ambulatory epileptic patients and 30 healthy matched subjects). The aim and procedures of the study were explained to every participant, and an informed consent was obtained before being enrolled in the study. The study was approved by the ethical committee at the Department of Neurology, Faculty of Medicine, Cairo University.

### Exclusion criteria included

The exclusion criteria included the following: age more than 50 years, history of previous fractures, family history of bone diseases, endocrinal disorders that may affect bone health (thyroid, parathyroid, and adrenal disorders), chronic illness that may affect bone health, patients receiving drugs that may affect bone health (corticosteroid therapy, chemotherapeutic agents, H2 blockers, or proton pump inhibitors), substance abuse, pregnant, lactating, and menopausal females.

### Methodology

- Measurements of bone health markers:
- Serum calcium and phosphorus by quantitative colorimetric method
- Serum alkaline phosphatase by enzyme-linked immunosorbent assay (ELISA)
- Serum parathormone by ELISA
- Serum 25 Vit D3 by ELISA
- Dual-energy X-ray absorptiometry (DEXA) scan: to assess bone mineral density

Measurement of bone mineral density (BMD) was performed at three sites: the lumbar spine, left wrist, and left hip. At the lumbar spine (LS), the patient lies supine on the imaging table and the legs are raised by support for the lower legs. At the left femur (LF), the patient lies supine on the imaging table where the legs are flat with feet strapped to a feet holder that position the measured leg at 300 inward rotation. At the left radius (LR), the patient sits beside the imaging table, with the arm positioned on the imaging table supported with a positioning device (Van der Sluis et al. 2002).

### The references used for BMD measurement

1. L2–L4 for the lumbar spine
2. Proximal femur for the left hip
3. Distal one third of radius for the left forearm

### Interpretation of results

BMD results were expressed in the form of *T* score which is the number of standard deviations below the peak bone mass in adult. *T* score above − 1 is considered normal, a score between − 1 and − 2.5 is classified as osteopenia, and a score below − 2.5 is classified as osteoporosis (Genant et al. 1999).

### Statistical analysis

The collected data were coded, tabulated, and statistically analyzed using IBM SPSS Statistics (Statistical Package for Social Sciences) software version 22.0, IBM Corp., Chicago, USA, 2013. Descriptive statistics were done for quantitative parametric data as minimum and maximum of the range as well as mean ± SD and for qualitative data as number and percentage. Inferential analyses were done for quantitative variables using independent *t* test in cases of two independent groups with parametric data and paired *t* test in cases of two dependent groups with parametric data and ANOVA test for more than two independent groups with parametric data, and then, post hoc test was used for pair-wise comparison based on Kruskal-Wallis distribution. Inferential analyses for qualitative data were done using chi-squared test for differences between proportions and Fisher’s exact test for variables with small expected numbers. Correlations were done using Pearson’s correlation for numerical parametric data. Logistic regression model was used to find out independent factors affecting certain conditions. The level of significance was considered as follows: *ρ* value < 0.05 is significant, *ρ* value < 0.01 is highly significant, and *ρ* value ≥ 0.05 is non-significant.

## Results

### General and disease characteristics of the patient group

The age of the patients ranged from 20 to 45 years with a mean age of 30.3 ± 8 years. Twenty patients (66.7%) were females, and ten patients (33.3%) were males.

Eighteen patients (60%) presented with focal epilepsy while 12 patients (40%) presented with generalized epilepsy.

According to Sweileh et al. (2011) who defined epilepsy as “well controlled” if the patient had no seizures for 3 months, 17 patients (56.7%) had uncontrolled seizures while 13 patients (43.3%) had controlled seizures.

Interictal EEG revealed that ten patients (33.3%) had focal epileptic discharge whereas six patients (20%) had generalized epileptic discharges.

Brain MRI was normal in 24 patients (80%), while six patients (20%) had abnormal MRI.

Eight patients (26.6%) received monotherapy: levetiracetam in one patient (3.3%), phenytoin in three patients (10%), and valproate in four patients (13.3%). Twenty-two patients (73.4%) received polytherapy: six patients (20%) were on enzyme inducers phenytoin and carbamazepine, five patients (16.7%) were on enzyme inhibitors valproate and levetiracetam, and 11 patients (36.7%) were on phenytoin and levetiracetam.

### Comparison analysis

#### Comparison between the studied groups regarding laboratory and radiological markers of bone health

There was a statistically highly significant difference between the two groups as regards serum laboratory markers (*ρ* value < 0.01) (Table 1).

**Table 1:** Range and mean values of bone health laboratory markers in the studied groups

Serum level	Patient group (*n* = 30)		Control group (*n* = 30)		*ρ* value
	Mean ± SD	Range	Mean ± SD	Range	
Calcium (mg/dl)	8.5 ± 0.5	7.5–9.5	9.1 ± 0.3	8.8–9.7	0.0001**
Phosphorus (mg/dl)	3.4 ± 0.6	2.7–4.9	4 ± 0.3	3.4–4.5	0.0001**
ALP (mg/l)	170.5 ± 43.9	102.6–246.6	96.1 ± 27.6	45.3–146.8	0.0001**
Parathormone (pg/ml)	85.2 ± 27.8	36.4–128.5	40.4 ± 12.2	22.3–62.3	0.0001**
Vitamin D (ng/ml)	21.8 ± 7.4	10.2–32.5	43.7 ± 7.6	28–56	0.0001**

*ALP* alkaline phosphatase

**ρ value < 0.01 = highly significant

Control subjects had *T* score > − 1. In the patient group: eight patients had normal results (*T* score > − 1), 17 patients had osteopenia (*T* score from − 1 to − 2.5), and five patients had osteoporosis (*T* score < − 2.5). The difference between patients and controls was statistically significant (*ρ* value = 0.0001).

**Fig. 1:**
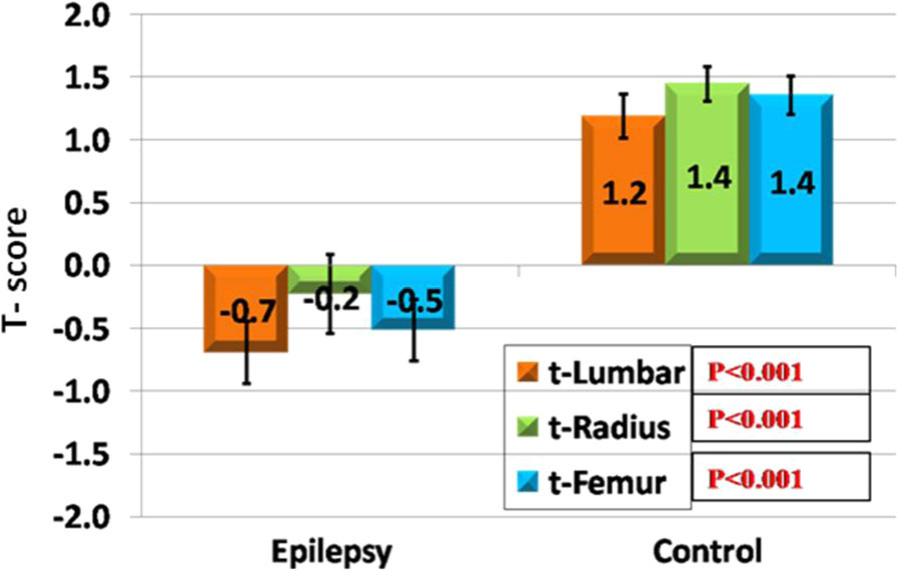
Comparison of the values of *T* scores at different regions between the studied groups

Bone mineral density and *T* score at the three regions (LS, LR, LF) were significantly lower in patients compared to controls (*ρ* value = 0.0001) (Fig. 1). Moreover, a statistically highly significant difference was detected in the mean values of BMD at the three regions in the patient group (*ρ* value < 0.01). Post hoc test revealed that BMD was lowest at the femur compared to the radius and lumbar regions (*ρ* value = 0.0001).

#### Comparison between patient subgroups (distributed according to the results of DEXA scan)

Epileptic patients were divided into three subgroups (normal, osteopenic, and osteoporotic) according to the results of DEXA scan.

There was a statistically significant difference between the patient subgroups as regards the mean age (*ρ* value = 0.039). Post hoc test revealed a significant difference between the normal and osteoporotic subgroups as regards the mean age being significantly younger in patients with normal DEXA scan (*ρ* value = 0.043).

A statistically highly significant difference was also found between patient subgroups as regards the duration of disease and therapy (*ρ* value = 0.0001). Post hoc test revealed that the duration of epilepsy and therapy was significantly longer in the osteoporotic subgroup compared to the other subgroups (*ρ* value = 0.0001).

No significant difference was found between patient subgroups as regards the age at onset of epilepsy, type of epilepsy, epilepsy control, or number of received AEDs.

Regarding laboratory markers of bone health, a statistically highly significant difference was detected between patient subgroups in serum levels of calcium, phosphorus, parathormone, and vitamin D (*ρ* value < 0.01) (Table 2).

**Table 2:** Comparison between patient subgroups regarding serum laboratory markers of bone health

Variables		Normal (a) (*N* = 8)	Osteopenia (b) (*N* = 17)	Osteoporosis (c) (*N* = 5)	*ρ* value	Pair-wise comparisons		
						(a) × (b)	(a) × (c)	(b) × (c)
Serum calcium (mg/dl)	Mean ± SD	8.9 ± 0.4	8.6 ± 0.4	7.8 ± 0.2	0.0001**	0.09	0.0001**	0.003**
	Range	8.2–9.5	8.0–9.2	7.5–8.0				
Serum phosphorus (mg/dl)	Mean ± SD	4.3 ± 0.4	3.2 ± 0.3	3.0 ± 0.3	0.0001**	0.0001**	0.0001**	0.2
	Range	3.7–4.9	2.8–3.9	2.7–3.5				
Alkaline phosphatase (IU/l)	Mean ± SD	153.4 ± 41.9	171.5 ± 46.6	194.4 ± 30.3	0.267	0.0001**	0.0001**	0.0001**
	Range	102.6–206.2	108.5–246.2	142.1–221.3				
Parathormone (ng/ml)	Mean ± SD	82.2 ± 19.7	82.5 ± 30.2	99.4 ± 31.2	0.0001**	0.95	0.0001**	0.0001**
	Range	50.7–105.2	36.4–114.2	51.4–128.5				
Vitamin D (nmol/l)	Mean ± SD	30.5 ± 2.3	20.7 ± 5.1	11.7 ± 1.2	0.0001**	0.0001**	0.0001**	0.0001**
	Range	27.2–32.5	13.1–29.2	10.2–12.9				

Subgroups: (a) normal, (b) osteopenia, and (c) osteoporosis

*Ρ* value > 0.05 = non-significant; **ρ value < 0.01 highly significant

#### Comparison between patients receiving enzyme inducer and enzyme inhibitor AEDs regarding laboratory and radiological markers of bone health

Epileptic patients receiving enzyme inducers had significantly lower serum calcium, phosphorus, and vitamin D compared to those receiving enzyme inhibitors. Moreover, epileptic patients receiving enzyme inducers had significantly lower BMD and *T* scores compared to those receiving enzyme inhibitors (Tables 3 and 4).

**Table 3:** Comparison between patients receiving enzyme inducers and enzyme inhibitors regarding laboratory markers of bone health

Serum level		Patients receiving enzyme inducers (*n* = 20)	Patients receiving enzyme inhibitors (*n* = 9)	*ρ* value
Calcium (mg/dl)	Mean ± SD	8.4 ± 0.5	8.9 ± 0.5	0.02*
	Range	7.5–9.2	8.0–9.5	
Phosphorus (mg/dl)	Mean ± SD	3.1 ± 0.3	4.0 ± 0.6	0.002**
	Range	2.7–3.9	3.0–4.8	
ALP (ug/ml)	Mean ± SD	177.8 ± 43.2	150.3 ± 42.5	0.123
	Range	108.5–246.2	102.6–214.6	
Parathormone (pg/ml)	Mean ± SD	87.0 ± 30.0	79.1 ± 23.6	0.493
	Range	36.4–128.5	45.8–114.2	
Vitamin D (ng/ml)	Mean ± SD	18.7 ± 6.1	27.9 ± 6.2	0.0001**
	Range	10.2–29.2	14.5–32.5	

*ALP* alkaline phosphatase

*Ρ* value > 0.05 = non-significant; *ρ value < 0.05 = significant; **ρ value < 0.01 highly significant

**Table 4:** Comparison between patients receiving enzyme inducers and inhibitors regarding *T* score results

*T* score		Patients receiving enzyme inducers (*n* = 20)	Patients receiving enzyme inhibitors (*n* = 9)	*P* value
Lumbar	Mean ± SD	− 1.1 ± 1.1	0.1 ± 1.5	0.0001**
	Range	− 3.0–1.2	− 1.9–2.7	
Radius	Mean ± SD	− 0.8 ± 1.5	1.0 ± 1.6	0.02*
	Range	− 2.9–2.7	− 1.8–3.5	
Femur	Mean ± SD	− 1.2 ± 0.8	0.8 ± 1.3	0.006*
	Range	− 2.6–0.9	− 1.7–3.0	

*Ρ* value > 0.05 = non-significant; *ρ value < 0.05 = significant; **ρ value < 0.01 highly significant

### Correlations

**Fig. 2:**
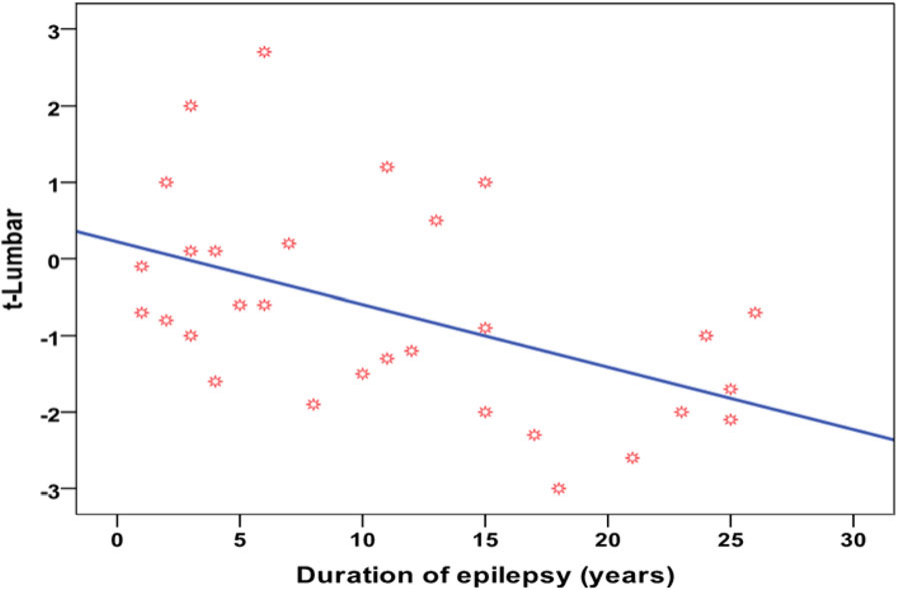
Correlation between *T* score of lumbar spine and duration of epilepsy and therapy in the patient group

A significant negative correlation was found between the duration of therapy and BMD and *T* score of lumbar spine, left radius, and left femur (Fig. 2).

BMD and *T* score at lumbar spine, left radius, and left femur were positively correlated with serum calcium, phosphorus, and vitamin D, while a significant negative correlation was found between these results at lumbar spine and serum alkaline phosphatase (Table 5).

**Table 5:** Correlation between *T* score results and laboratory markers of bone health among patient groups

Serum level	Correlation coefficient	*T-*lumbar	*T-*radius	*T-*femur
Calcium (mg/dl)	*r*	0.4	0.5	0.56
	*P*	0.02*	0.004**	0.001**
Phosphorus (mg/dl)	*r*	0.637	0.56	0.75
	*P*	0.0001**	0.001**	0.0001**
Alkaline phosphatase (IU/l)	*r*	− 0.47	− 0.3	− 0.26
	*P*	0.009**	0.1	0.2
Parathormone (ng/ml)	*r*	− 0.2	− 0.2	− 0.07
	*P*	0.3	0.4	0.7
Vitamin D (nmol/l)	*r*	0.653	0.84	0.84
	*p*	0.0001**	0.0001**	0.0001**

*P* value > 0.05 = non-significant; *ρ value < 0.05 = significant; **p value < 0.01 highly significant

### Logistic regression analysis

A logistic regression model was done to estimate the ODD ratio for BMD abnormality (osteopenia/osteoporosis) from independent risks. All risk factors were analyzed like age, sex, type of epilepsy, degree of disease control, age of disease onset, duration of disease and therapy, laboratory markers (Ca, PO4, ALP, PTH, Vit D), and DEXA scan results (BMD, *T* scores in lumbar spine, left femur, left radius). But we will not able to analyze each drug separately due to small number of cases on each drug as well as there were many cases on drug combination. It was that the duration of therapy significantly increases the risk of BMD abnormality (osteopenia/osteoporosis) annually by 1.366 in comparison to non-epileptic subjects, as well as the risk for BMD abnormality mildly increases in females by 0.118 in comparison to males, while other risk factors have no significant effect on BMD abnormality (osteopenia/osteoporosis).

## Discussion

Several large epidemiological studies indicated that patients with epilepsy are at two- to sixfold increase in the risk of fractures compared to the general population (Souverein et al. 2005; Vestergaard et al. 1999; Espallargues et al. 2001; Mattson and Gidal 2001). Epilepsy increases the risk for fracture by a variety of mechanisms. Accumulating evidence indicated that antiepileptic drugs (AEDs) are major risk factors for bone disease in non-institutionalized patients and well-nourished ambulatory adults with epilepsy (Boluk et al. 2004; Sheth et al. 2008).

This study revealed a statistically significant difference in the serum level of laboratory markers between epileptic patients and normal subjects. Serum calcium, phosphorus, and vitamin D were significantly lower, whereas serum alkaline, phosphatase, and parathormone were significantly higher in patients compared to controls. This goes in agreement with most previous studies (Oner et al. 2004; Hamed et al. 2014; Pack 2004; Mintzer 2010; Verrotti et al. 2010).

In contradistinction, Verrotti et al. (2002) found no significant reduction in vitamin D level with the use of AEDs. The studies done by Verrotti et al. (2000, 2002) also found no significant differences in PTH levels between epileptic patients on AEDs and normal population, which may be attributed to the inclusion of younger patients with a mean age around 20 who were on AEDs for a mean duration of 2 to 3 years. Many studies reported that the pathological effects of AEDs start to appear mainly after 5 years of AED treatment (Oner et al. 2004; Feldkamp et al. 2000).

In the present study, bone mineral density and *T* scores were significantly lower in epileptic patients compared to normal subjects, a finding which agreed with many epidemiological studies link AED use to decreased bone mass in both sexes. (Hamed et al. 2014; Petty et al. 2005; Kishk et al. 2008; Shaker et al. 2009) AEDs were found to increase vitamin D catabolism through the orphan (PXR) nuclear receptor resulting in increasing bone turnover (Pascussi et al. 2005). They have also direct effect on bone cells. Enzyme inducers have been demonstrated to inhibit the proliferation of human osteoblast-like cell at concentrations equal to therapeutic doses (Feldkamp et al. 2000) while hepatic enzyme inhibitor has been reported to act by stimulating osteoclast activity causing imbalance between bone formation and resorption, contributing to bone loss (Sheth et al. 1995).

The current study revealed that BMD was lowest at the femur compared to the radius and lumbar regions. This was in accordance with previous studies that found an increased risk for pathological fracture in epileptic patients in comparison to general population mainly in the femur. The femur is the strongest weight-bearing bone that contains large number of cells and huge amount of bone matrix, so it is the most affected bone by AEDs (Souverein et al. 2005; Mattson and Gidal 2001; Sheth et al. 2008).

A significant difference was found between normal and osteoporotic patients as regards the mean age being significantly younger in patients with normal DEXA scan. However, no significant correlation was found between the mean of age of epileptics and BMD and *T* scores because we selected patients less than 55 years old to minimize the effect of age on bone loss. This agreed previous studies done by Kishk et al. (2008) and Shaker et al. (2009). On the other hand, Petty et al. (2005) found significant negative correlation between the mean of age of epileptics and DEXA scan results in old epileptic greater than 40 years.

A significant negative correlation was found between the duration of AED therapy and BMD as well as *T* scores; this was in agreement with most of the previous studies (Oner et al. 2004; Hamed et al. 2014; Canevini et al. 1995; Stephen and Brodie 2012). On the contrary, Kishk et al. (2008) failed to find significant correlation between duration of disease and DEXA scan results, which may be attributed to patient selection as the mean age of studied group was 26 years, with a mean disease duration of 5 years.

No significant relation was detected between the type of epilepsy and abnormalities in DEXA scan (*ρ* = 0.18). This finding disagreed with the study done by Farhat et al. (2002) who found that generalized epilepsy is significantly associated with abnormal DEXA scan results. The contradistinction may be attributed to patient selection, as we included 18 patients out of 30 with focal fits, while Farhat et al. (2002) included a large number of patients with generalized epilepsy taking high doses of AEDs as well as dual and triple therapy and a small number of patients with focal fits.

In the current study, we found no significant difference between patient subgroups as regards the number of received AED. Our results did not concord with many studies that found significant negative correlation between the number of received AEDs and BMD (Oner et al. 2004; Feldkamp et al. 2000; Farhat et al. 2002). These studies were done on hundreds of patients receiving multiple AEDs for a mean duration around 10 years.

Patients receiving enzyme inducers had significantly lower serum calcium, phosphorus, and vitamin D compared to those receiving enzyme inhibitors. This agreed with the studies done by Ali et al. (2004) and Pack et al. (2004) who found decrease serum calcium and phosphorus in patients receiving enzyme inducers than those receiving inhibitors. Other studies also revealed low levels of biologically active vitamin D in patients particularly those receiving enzyme inducers than those receiving inhibitors (Feldkamp et al. 2000; Farhat et al. 2002).

Enzyme inducers have several mechanisms which affect vitamin D, calcium, and phosphorus metabolism than enzyme inhibitors. They induce CYP 450 and pregnane X receptor (PXR) activation which in turn increases catabolism of vit. D, with subsequent decrease in serum calcium and phosphorus level as well as secondary hyperparathyroidism. This increases bone turnover with subsequent increase in alkaline phosphatase level as a marker of bone resorption (Valsamis et al. 2006). On the contrary, Kafali et al. (1999) failed to find significant decrease in serum calcium and phosphorus levels with the use of enzyme-inducing AEDs in comparison to enzyme inhibitors. This study was done on 60 epileptic patients on AEDs with a mean duration around 2 years where the adverse effect of AEDs on bone metabolism had not established yet.

No significant difference was found between enzyme inducer and enzyme inhibitor groups regarding serum alkaline phosphatase and parathormone. This finding disagreed with the studies done by Erbayat et al. (2000) and Ecevit et al. (2004) who found high PTH level in patients receiving enzyme inducers than those receiving enzyme inhibitors. The contradistinction may be attributed to the inclusion of children and young adults in these studies, in whom there is continuous dynamic nature of BMD (Beerhorst et al. 2005).

Epileptic patients receiving enzyme inducers had significantly lower lumbar, radius, and femur BMD and *T* scores compared to those receiving enzyme inhibitors. This was in agreement with the many studies that found significant decrease in bone mineral density in epileptic patients receiving enzyme inducers than those receiving inhibitors (Dennis et al. 2005; Arora 2007). This was also in accordance with the studies done by Canevini et al. (1995) and Stephen and Brodie (2012) who found that the fracture risk is associated with the use of enzyme inducer like phenytoin (DPH), carbamazepine (CBZ), phenobarbital (PB), oxcarbazepine (OXC), and clonazepam (CZP). This is because enzyme inducers have several mechanisms which affect BMD than enzyme inhibitors. They have direct effect on bone cells as well as indirect effect through modulation of hormones affecting bone metabolism (Valsamis et al. 2006).

In this study, a significant positive correlation was detected between the BMD as well as *T* scores and (serum calcium, phosphorus, and vitamin D). This was in accordance with the study done by Hamed et al. (2014). In contradiction, Feldkamp et al. (2000) and Farhat et al. (2002) found no significant correlation with DEXA scan results although they found low levels of vitamin D, calcium, and phosphorus with AED use.

Serum alkaline phosphatase was negatively correlated with BMD and *T* score of lumbar spine. This disagrees with the studies done by Oner et al. (2004), Verrotti et al. (2002), and Shaker et al. (2009), although they reported an increase level of alkaline phosphatase in epileptic patients on AEDs. This may be due to the fact that ALP is not sensitive enough to detect changes in bone remodeling seen in most cases of uncomplicated osteoporosis (Valimaki et al. 1994). No significant correlation was found between serum parathormone and BMD as well as *T* scores. This was in accordance with the studies done by Pack and Morrel (2004), Mintzer (2010), Verrotti et al. (2010), and Ali et al. (2004) who failed to find a significant correlation between serum parathormone and DEXA scan results although high levels were detected in epileptic patients.

Logistic regression analysis revealed that the duration of disease and therapy significantly increases the risk of BMD abnormality annually by 1.366 in comparison to non-epileptic subjects. This finding was in agreement with that of Dennis et al. (2005) who found a significant increase in the risk of BMD abnormality with the duration of AED therapy whatever the type of drug used. It was found also that the risk of BMD abnormality mildly increases in females by 0.118 in comparison to males. This was also in accordance with the studies done by Valimaki et al. (1994) and Stephen et al. (1999) who found an increased risk for BMD abnormalities in females.

## Conclusions

In view of the results of this study, we can conclude that abnormalities of bone health are common among epileptic patients. These abnormalities can be attributed mainly to the prolonged usage of AEDs, especially enzyme inducers. So, prophylactic supplementation of calcium and vitamin D is recommended in patients receiving AEDs in order to decrease the risk of bone disease in such patients.

We recommend the use of objective scale for assessment of physical activity.

We could not assess the frequency of no traumatic fractures in epileptic patients as history of previous fracture was an exclusion criterion. So, further studies are warranted to assess the risk of no traumatic fractures with AEDs.
